# Rehabilitation for cancer patients at Black Lion hospital, Addis Ababa, Ethiopia: a cross-sectional study

**DOI:** 10.1186/s12904-017-0235-7

**Published:** 2017-11-16

**Authors:** Teshager Worku, Zuriash Mengistu, Agumasie Semahegn, Gezahegn Tesfaye

**Affiliations:** 1 College of Health and Medical Sciences, Haramaya University, PO. Box 235, Harar, Ethiopia; 2 School of Allied Health, Department of Nursing and Midwifery, College of Health Science, Addis Ababa University, Addis Ababa, Ethiopia

**Keywords:** Cancer rehabilitation service, Utilization, Barriers, Black lion hospital, Ethiopia

## Abstract

**Background:**

In Ethiopia, there were greater than 2000 adult and 200 pediatric cancer patients annually in 2010, but the estimated number of cancer patients were increasing. Oncologic rehabilitation treatment may result in improved physical and mental impairment. There is a paucity of information about rehabilitation service utilization among cancer patients in Ethiopia. Hence, the purpose of this study was to assess the rehabilitation service for cancer patient and associated factors at Black Lion hospital, Addis Ababa, Ethiopia.

**Methods:**

A hospital-based cross-sectional quantitative study was conducted from March to April 2014. Convenient sampling method was employed to recruit the study participants. Interviewer administered questionnaire was used to collect data. Data were entered into EPI data version 3.1 and exported to SPSS (16.0) software for analysis. Descriptive analysis, binary and multiple logistic regression were carried out. Significance association was interpreted using adjusted odds ratio at 95% confidence interval and *p*-value less than 0.05.

**Result:**

A sample of 423 patients aged 18 years and older were involved in the study. Breast cancer (25%), colorectal cancer (20.6%), cervical cancer (14.7%), lymphoma (7.7%), lung (7.2%), leukemia (5.4%), kidney (3.6%) and prostate cancer (2.6%) were the common forms of cancer diagnosed at cancer unit of the Black Lion Hospital. Twenty six percent of cancer patients received rehabilitation service at least once. The main rehabilitation services given were nutritional and psychological support. Unavailability of supplies, lack of professionals and cost of service were among the barriers to receiving rehabilitation services.

**Conclusion:**

Only a few cancer patients received cancer rehabilitation services. Increasing the knowledge of the professionals, stocking cancer units with necessary supplies, and other comprehensive programs are needed.

## Background

Globally, cancer has become a major public health tricky and an increasingly important contributor to the burden of diseases [1]. Cancer causes an estimated 12.7 million new cases, 28 million chronic cases and 7.6 million deaths within five years from the initial diagnosis in 2011 [1, 2]. In the United States, an estimated 569,490 deaths from cancer occurred in 2010. Although the incidence of cancer is increasing, improvements in early diagnosis and treatment have led to significantly increased survival rates in recent years [3, 4]. The number of cancer survivors has exceeded 11 million and continues to grow. An advanced form of cancer is often accompanied by significant symptom, psychosocial distress and poor quality of life [5, 6]. Unfortunately, cancer treatments may result in physical and mental impairment such as dysfunction of the nerve, musculoskeletal and internal organ systems. Cancer-related fatigue and deconditioning have also been frequently reported as side effects of the treatments. These all contribute to the impairments and loss of functions [7, 8].

These ongoing problems faced by the patients upsurge the need for rehabilitation service. Several studies have shown that rehabilitation can alleviate post-treatment side effects, maintain quality of life and improve the survival rate [9, 10]. Rehabilitation refers to a process aimed at enabling persons with disabilities to achieve and maintain their optimal physical, sensory, intellectual and or social functional levels to adapt their lives toward a higher level of independence [11]. The rehabilitation service includes preventive, restorative, supportive and palliative rehabilitation therapy [12]. Rehabilitation services is underutilized by cancer patients due to various barriers such as lack of access and readiness to utilize, expense, time limitations, difficulty in obtaining transportation, lack of knowledge and poor referral linkage [7, 8, 13].

Cancer rehabilitation is a relatively new area of research in Ethiopia, according to data from the Black Lion Hospital cancer unit registry, in 2014 a total of 2040 cancer patients have received cancer rehabilitation care, indicating that the rehabilitation service need is growing. We could not find adequate published studies on cancer rehabilitation care and support service utilization in Ethiopia. This highlight the need to conduct further studies in the area to provide information for planners, programmers and policy makers. Therefore, we aimed to assess the cancer rehabilitation service and associated factors among cancer patients at Black Lion Hospital, Addis Ababa, Ethiopia.

## Methods

### Study area and period

This study was conducted at the cancer unit of the Black Lion Hospital in Addis Ababa, Ethiopia from March to April 2014. Addis Ababa is the capital city of Ethiopia, and the political capital of African union and other international organizations. According to the central statistics agency report of census 2007, Addis Ababa city has a total population of 3,384,569. Black Lion hospital is the biggest referral public hospital in Ethiopia. Black Lion Hospital is the training center health professionals including undergraduate and postgraduate medical students, dentists, nurses, pharmacists, laboratory technicians and others paramedics. The hospital was staffed by many health professionals from various disciplines including physicians, nurses, oncology nurses, medical oncologists, specialized surgical oncologist, pathologists, hematologists, radiotherapists, pediatric oncologist, general and specialist surgeons, CT and MRI scanner and cobalt radiotherapy unit. In 2014, the hospital has total beds of 678 and the bed reserved for cancer care at oncology unit was 20, The cancer unit of the black lion hospital has provided chemotherapy, radiation therapy, complain therapy and other supportive and palliative cares. It is the main center for cancer registry, early detection, prevention, standard treatment and palliative care in Addis Ababa.

### Study design and participants

A hospital-based cross-sectional quantitative study was conducted. Patients aged 18 years or older who had diagnosed with any type of cancer during the data collection period were included in the study. Meanwhile, those patients who were newly diagnosed, critically ill, have known hearing problem and or cognitively impaired to give consent were excluded.

### Sample size determination and sampling technique

The sample size was determined using single population proportion formula (*n* = (Z α/2) ^2^ p (1-p)/d^2)^; where as, *n* = sample size, Zα/2(1.96): significance level at α =0.05, P: expected proportion of adult cancer patients’ who utilize rehabilitation service (50%), d: margin of error (0.05) and 10% non-response rate. The final sample size was 423. We used convenience sampling method to recruit study participants due to hardly nature to construct sampling frame because of unpredictable number of patients who were coming from different units and referral for combined therapy.

### Measurements and data collection techniques

A 29-item structured cancer rehabilitation program questionnaire from available literature [14] was adapted and modified to suit to the study objective. The questionnaire consisted of socio-demographic and economic factors (age, sex, ethnicity, religion, level of education, type of occupation, house hold income, social status), availability of cancer rehabilitation service, history of cancer in the family, information about cancer, accessibility of rehabilitation service, cost, social and family support, and availability of professionals trained on cancer rehabilitation. The questionnaire was translated to the local language (Amharic), and re-translated to English for consistency. Patients were selected by cancer care unit in-charge based on the inclusion criteria. The data collectors interviewed the cancer patients in quiet and confidential place. The data were collected by cancer unit nurses.

### Data quality assurance and management

The data collectors and supervisors were trained prior to the actual conduct of the data collection about purpose of the study, sampling procedure, methods of data collection, ethical issues and ways of addressing contingency management skills. Prior to data collection, a pretest was conducted on 5% of cancer patients to check the consistency and appropriateness of the questionnaire. Then necessary revisions were undertaken prior to the actual data collection. Two Bsc nurses daily supervised the data collection process. The questionnaires were reviewed and checked for completeness, accuracy and consistency. Necessary and timely corrective measures were taken.

### Data processing and analysis

The collected data were checked for completeness, cleaned and entered into EPI data version 3.1, and exported to SPSS version 16.0 software for analysis. The data were cleaned by running descriptive (frequency) to explore some data anomalies and outliers. Descriptive statistical analysis was used to compute frequency, percentages, mean and standard deviations. Binary and multiple logistic regressions were carried out to examine the relationship between cancer rehabilitation service utilization and its associated factor. Statistical association was declared using adjusted odds ratio at 95% confidence interval and *p* value less than 0.05.

## Results

A total of 423 respondents were involved in the study with a response rate of 91.7%. As a result, 388 respondent’s data was included in the analysis.

### Socio-demographic characteristics

More than one-thirds, 68.6% (266/388) of respondent were females. Majority of the cancer patients belonged in the age (18–39 years), 41.8% (162/388). The mean age was 44(±14.9) years. Sixty two percent (241/388) were Orthodox Christianity followers 62.1%, 58% (225/388) were married and 41% (159/388) belonged to Amhara ethnic (Table 1).

**Table 1:** Socio-demographic characteristics of adult cancer patients at cancer center of Black Lion hospital, Addis Ababa, Ethiopia, in April 2014 (*n* = 388)

Variable	Category	Frequency	Percent
Sex	Male	122	31.4
	Female	266	68.6
Age	18–39	162	41.8
	40–49	85	21.9
	50–59	76	19.6
	60–69	47	12.1
	70–79	11	2.8
	80+	7	1.8
Religion	Orthodox	241	62.1
	Muslim	86	22.2
	Protestant	42	10.8
	Catholic	13	3.4
	Others	6	1.5
Marital status	Married	225	58
	Single	94	24.2
	Widowed	41	10.6
	Divorced	28	7.2
Ethnicity	Amhara	159	41
	SNNP	88	22.7
	Oromo	56	14.4
	Tigre	55	14.2
	Other	30	7.7
Occupation	Governmental employee	77	19.8
	House wife	67	17.3
	Farmer	28	7.2
	Merchant	34	8.8
	Student	24	6.2
	Private or NGO	19	4.9
	Retired	25	6.4
	Stopped	105	27.1
	Others	9	2.3
Educational status	Read and write but no formal education	46	11.9
	Primary education (1–6)	44	11.3
	Secondary education (7–12)	90	23.2
	Tertiary education (12+)	117	30.2
Monthly income (ETB)	<313.75	97	25.0
	313.75–900	103	26.5
	900–2330	91	23.5
	>2330	97	25.0

### Information about cancer

More than one-fourth 27.6% (107/388) of cancer patients had got information about cancer. Of these, health institution (41.7%) was the main source of information followed mass media (television, 26.1%). About 5.7% (22/388) of them encountered with other types of cancer previously. On the other hand, 4.9% of the respondents involved in care of patients with cancer, while 8.8% respondents knew someone with cancer having different kind of relationship with him/her.

### Types of cancer diagnosis

The most common cancer diagnosis seen at the center was breast cancer, 25% (97/388). Other type of cancer was colorectal cancer 20.6% (80/388), cervical cancer 14.7%(57/388), Lymphoma 7.7%(30/388), Lung 7.2%(28/388), Leukemia 5.4%(21/388), Kidney (3.6%, 14/388) and prostate cancer 2.6%(10/388). majority of the respondents 62.6%(243/388) described the stages of illness as localized types of cancer, while 36.3% (141/388) described that their stages of illness is disseminated, two study participants reported having both disseminated and localized stages of cancer, and another two participants explained that they did not know the stage of cancer.

### Types of rehabilitation service received

Twenty six percent (101/388) of patients with cancer received the rehabilitation service at least once. Of these, almost half (49.5%, 50/101) of the patients received nutritional support, followed by psychosocial support 40.6% (41/101), Lower extremities (1%), balance training (1%), body awareness and flexibility (1%) (Table 2).

**Table 2:** Types of rehabilitation service rendered at Black Lion Hospital, Addis Ababa, Ethiopia in April 2014 (*n* = 388) (multiple responses were possible)

Types of rehabilitation service	Category	n(%)
Walking	Yes	6(6.0)
Upper extremity strength exercise	Yes	8(8.1)
Lower extremities strength exercise	Yes	1(1.0)
Balance training	Yes	1(1,0)
ADL training (toileting, grooming)	Yes	3(3.0)
Self-management (care for her/himself)	Yes	3(3.0)
Energy conservation(positioning, environment adjustment)	Yes	3(3.0)
Nutritional support (nutritional counseling)	Yes	50(49.5)
Psychological support	Yes	41(41.4)
Relaxation training (recreational, massage)	Yes	4(4,.0)
Body awareness /body image	Yes	1(1,0)
Stress management	Yes	2(2.0)
Flexibility exercise	Yes	1(1.0)
Education rehabilitation	Yes	38(38.4)
Treatment of side effect	Yes	16(16.2)
Symptom treatment	Yes	16(16.2)
Other	Yes	3(3.0)

### Health education rehabilitation service

Out of the overall respondents who are getting educational rehabilitation, 78.7% (59) got nutritional counseling followed by pain management 35.1% (26), symptom treatment 17.6% (13), family education 16.2% (12), strength exercise 12.2% (9) and energy conservation 5.4% (4), respectively (Table 3). With regards to the methods of education, out of the total participants who got educational rehabilitation, 77.3% (58) got education by one to one discussion and 24% (18) got in group discussion, the rest of the participants got education by other means 4% (3) and 65.3% (49) of them got education through discussion with families.

**Table 3:** Involvements in the education program rehabilitation at Black Lion Hospital, Addis Ababa, Ethiopia, in April 2014 (*n* = 388)

Education	Category	N(%)
	Yes	72 (73.5)
Aerobic exercise	Yes	2 (2.7)
Nutritional support	Yes	59(78.7)
Strength exercise	Yes	9(12.2)
Relaxation exercise	Yes	4(5.4)
ADL/feeding washing	Yes	4(5.4)
Energy conservation	Yes	4(5.4)
Pain management	Yes	26(35.1)
Travel	Yes	2(2.7)
Sleeping technique	Yes	5(6.8)
Complementary treatment (acupuncture)	Yes	1(1.4)
Symptom treatment	Yes	13(17.6)
Family education	Yes	12(16.2)
Other method	Yes	2(2.7)

### Type health professionals who provide the rehabilitation service

**Fig. 1:**
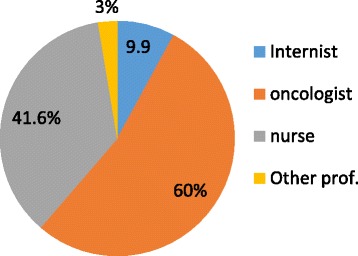
Health professionals’ involvement at cancer center of Black Lion hospital, Addis Ababa, Ethiopia, in April 2014 (*n* = 388)

Approximately one-third 60.4% (61/101) of cancer patients were provided with the rehabilitation service by oncologist. While 41.6% (42/101) of them received the rehabilitation service by nurse professionals and the remaining 9.9% (10/101)) obtained rehabilitation service from medical internist (Fig. 1).

### Barriers of rehabilitation service utilization

Approximately one-fourth (23.2%, 90/388) were satisfied with the cancer rehabilitation service. The most common barriers for not receiving the cancer rehabilitation service were lack of availability of adequate space 60.7% (181/298), cost 28.9% (86/298), inaccessibility 46.6% (124/298), lack of health care professionals with experience in cancer care 50.3% (150/298), lack of support 12.8% (37/298), lack of awareness 42.9% (128/298), lack of knowledge of family members 40.9% (122/298), failure to identify acute illness 6.4% (19/298) and lack of referral 17.4% (52/298) (Table 4).

**Table 4:** Barriers of rehabilitation service utilization at cancer center of Black Lion hospital, Addis Ababa, Ethiopia, April 2014

Barriers	Category	Frequency	Percent
Unavailability	Yes	181	63.3
	No	105	36.7
Lack of professionals	Yes	150	49.3
	No	154	50.7
Cost	Yes	86	28.4
	No	216	71.3
Inaccessibility	Yes	124	46.8
	No	141	53.2
Lack of support	Yes	37	14.1
	No	226	85.9
Lack of resource	Yes	14	5.5
	No	242	94.5
Dissatisfaction with the program	Yes	11	4.3
	No	244	95.7
Acute illness	Yes	8	3.1
	No	247	96.9
Side effect of treatment	Yes	11	4.3
	No	244	95.7
Musculoskeletal injury	Yes	9	3.5
	No	246	96.5
Hopelessness	Yes	12	4.6
	No	248	95.4
Lack of referral	Yes	52	19.8
	No	211	80.2
Lack of awareness	Yes	128	44.6
	No	159	55.4
Lack of knowledge of family members Failure to identify acute illness	Yes	112	40.6
	No	164	59.4
	Yes	19	87.4
	No	239	92.6
Other	Yes	25	6.5
	No	362	93.5

### Factors associated with rehabilitation service utilization

After adjusting for all the predictor variables, knowing someone with cancer, lack of support, lack of professionals, lack of awareness, unavailability of the service and lack of knowledge were significantly associated with rehabilitation service utilization. Cancer patients who know someone with cancer have a higher odds of utilizing cancer rehabilitation service (AOR, 3.54; 95% CI: 1.08, 11.6) compared to patients who did not know any one. Those patients who reported that there was no availability of rehabilitation service were less likely to use cancer rehabilitation service (AOR, 0.15; 95% CI: 0.70, 0.32) when compared with those patients reported as there was availability of the service. Those participants with lack of appropriate professionals was less likely to use rehabilitation service (AOR, 0.35; 95% CI: 0.14, 0.85**)** compared with those with appropriate professionals**.** Respondents having lack of support were less likely to utilize rehabilitation service (AOR, 0.07, 95% CI: 0.07, 0.66) when compared with respondents with good support. Patients who have lack of awareness were less likely to utilize rehabilitation service (AOR, 0.32, 95% CI: 0.13, 0.81) that those who have good awareness. Those patients who have poor level of knowledge among the family members are also less likely to use rehabilitation service (AOR, 0.36, 95% CI: 0.13, 0.99) when compared with those respondents with good knowledge among family members (Table 5).

**Table 5:** The association between independent variables and cancer rehabilitation service utilization, at cancer center of Black Lion hospital, Addis Ababa, Ethiopia, April 2014

Variable	Utilization of rehabilitation service					
		Yes	No	COR (95% CI)	AOR (95% CI)	*P* value
Cancer Information in the past 1 year	Yes	41(10.6)	66(17)	2.29(1.42–3.71)		
	No	60(15.5)	221(57)	Ref		
Know someone with cancer	Yes	16(4.1)	18(4.6)	**2.81(1.37–5.76)**	**3.54(1.08–11.61)**	0.037*
	No	85(21.9)	269(69.3)	Ref	Ref	
Involvement in care of cancer patient	Yes	10(2.6)	9(2.3)	3.39(1.34–8.61)		
	No	91(23.4)	278(71.6)	Ref		
Unavailability of service	Yes	16(4.1)	165(42.5)	**0.11(0.06–0.20)**	**0.15(0.70–0.32)**	0.000*
	No	50(13)	55(14.1)	Ref	Ref	
Lack of professionals	Yes	8(2)	142(36.6)	**0.09(0.04–0.20)**	**0.35(0.14–0.85)**	0.020*
	No	58(14.9)	96(24.7)	Ref	Ref	
Inaccessibility of the service	Yes	15(3.8)	109(28)	0.24(0.13–0.46)		
	No	51(13.1)	90(23.2)	Ref		
Lack of support	Yes	1(0.25)	36(9.27)	**0.070(0.01–0.52)**	**0.07(0.07–0.66)**	0.020*
	No	64(16.5)	162(41.7)	Ref	Ref	
Lack of referral	Yes	7(1.8)	45(11.6)	6.40(0.171–0.94)		
	No	59(15.2)	152(39.14)	Ref		
Lack of awareness	Yes	9(2.3)	119(30.7)	**0.14(0.06–0.29)**	**0.32(0.13–0.81)**	0.016*
	No	57(14.7)	102(26.3)	Ref	Ref	
family members Lack of knowledge	Yes	9(2.3)	103(26.5)	**0.16(0.08–0.35)**	**0.36(0.13–0.99)**	0.047*
	No	57(14.7)	107(27.6)	Ref	Ref	

*P*-value < 0.05 significant, written with bold and in star (*) sign shows significant factors for multivariate analysis

## Discussion

This study determined the level of cancer rehabilitation care and support service utilization and associated factors at Black Lion Hospital, Ethiopia. The overall rehabilitation service utilization among cancer patients was 26%. Types of rehabilitation service obtained by the patients were nutritional support (49.5%), psychological support (41.1%), education rehabilitation (38.1%), upper extremities strengthening exercises (8.1%) and walking skill training (6%). Knowing someone with cancer, unavailability of service, lack of support, lack of awareness, lack of professionals, lack of referrals, lack of knowledge among family members were significantly associated with rehabilitation service utilization.

One in four (26%) of study participants received rehabilitation service at least once. Meanwhile, those cancer patients who have utilized cancer rehabilitation service were satisfied by the care and support service received. This finding is lower than a study conducted in Denmark [15] which reported that 52% of the cancer patients had participated in at least one rehabilitation activity. However, this finding is a bit higher than a study done in Taiwan [16] which indicated that 12.8% of the cancer patients received the care and support service. The possible explanation for the discrepancy might due to the fact that there are difference in the context of the country which results in difference in the health care delivery system, and availability of health infrastructure to provide rehabilitative services and availing trained health workers.

Knowing someone with cancer was positively associated with cancer rehabilitation service utilization. Meeting or knowing someone from families, relatives, friends who might have cancer can have positive influence on the patient motivation and interest to use the service. Lack of awareness, lack of professional, lack of support and lack of referral linkage were also statistically significant factors that decrease the rehabilitation service utilization. This study finding is consistent with other study [17], a study in Denmark [15], a study done in Canada [14] and a study done in Japan [18] revealed that study done in lack of awareness of rehabilitation services, and lack of knowledge among family members, lack of support, a failure of acute-care staff to identify functional impairments, lack of appropriate rehabilitation referral, lack of awareness of rehabilitation services, and affect rehabilitation service utilization.

This study revealed that almost one third (35%) of cancer patients experienced functional loss due to physical weakness, and required assistance with performance on Activity of Daily Living (ADL). This finding is in lined with other studies [19–21] revealed that patients experienced difficulty with ambulation (23%), and had deficits in transfers (7%). an effort to improve the quality of life of cancer survivors increasing attention has been given to improving functional recovery following treatment. Rehabilitation has been proposed as a strategy for restoring patients’ functional independence and improving their psychological function. Another study done in Taiwan [22] revealed that most rehabilitation therapy occurred as an outpatient service (96.0%), physical therapy (84.2%), occupational therapy (15.4%), physical therapy moderate degree (60.5%), physical therapy complicated degree (16.2%), and speech/swallowing therapy (0.4%) were the most commonly used programs.

### Strength and limitation of the study

Strength of the study was that the study tool was developed from previously used standardized and piloted instrument for measuring rehabilitation service utilization. The study was conducted on new area of care so that it can help further studies at national level to build upon on this finding. Since the study design was cross sectional study design it was not possible to establish temporal relation between the exposure and outcome variable. The result may not representative of entire cancer patients in Ethiopia. Finally the information was obtained through interviewer administered questioner so that response was prone to social desirability bias.

## Conclusion

Breast, colorectal and cervical cancers were the most commonly seen cancer on patients attending the oncology unit of black lion hospital. Only one-fourth of the patients with cancer received rehabilitation service at least once. The most widely used type of rehabilitation service was nutritional support. Knowing someone with cancer, unavailability of services, lack of professional, lack of support, lack of awareness, and lack of knowledge among family members were significantly associated with poor utilization of cancer rehabilitation service. We recommend that interventions should be carried out to enhance coordinated cancer rehabilitation service delivery to address the wide range of psychological, nutritional, social support, education and also train health professionals on the rehabilitation service provision. Further research should be conducted on rehabilitation service utilization and its determinants in Ethiopia.

## Abbreviations

ADL: Activity of daily living
ETB: Ethiopian Birr
FMOH: Federal Ministry of Health
NGO: Non-Governmental Organization
SPCS: Supportive and palliative care service
SPSS: Statistical package of social sciences
WHO: World Health Organization
